# B Cell and CD4 T Cell Interactions Promote Development of Atherosclerosis

**DOI:** 10.3389/fimmu.2019.03046

**Published:** 2020-01-10

**Authors:** Christopher Tay, Peter Kanellakis, Hamid Hosseini, Anh Cao, Ban-Hock Toh, Alex Bobik, Tin Kyaw

**Affiliations:** ^1^Vascular Biology and Atherosclerosis Laboratory, Baker Heart and Diabetes Institute, Melbourne, VIC, Australia; ^2^Centre for Inflammatory Diseases, Department of Medicine, Faculty of Medicine, Nursing and Health Sciences, Monash University, Melbourne, VIC, Australia; ^3^Department of Immunology and Pathology, Faculty of Medicine, Nursing and Health Sciences, Monash University, Melbourne, VIC, Australia

**Keywords:** atherosclerosis, B cells, CD4 T cells, MHCII, CD40

## Abstract

Interaction between B and CD4 T cells is crucial for their optimal responses in adaptive immunity. Immune responses augmented by their partnership promote chronic inflammation. Here we report that interaction between B and CD4 T cells augments their atherogenicity to promote lipid-induced atherosclerosis. Genetic deletion of the gene encoding immunoglobulin mu (μ) heavy chain (μMT) in ApoE^−/−^ mice resulted in global loss of B cells including those in atherosclerotic plaques, undetectable immunoglobulins and impaired germinal center formation. Despite unaffected numbers in the circulation and peripheral lymph nodes, CD4 T cells were also reduced in spleens as were activated and memory CD4 T cells. In hyperlipidemic μMT^−/−^ ApoE^−/−^ mice, B cell deficiency decreased atherosclerotic lesions, accompanied by absence of immunoglobulins and reduced CD4 T cell accumulation in lesions. Adoptive transfer of B cells deficient in either MHCII or co-stimulatory molecule CD40, molecules required for B and CD4 T cell interaction, into B cell-deficient μMT^−/−^ ApoE^−/−^ mice failed to increase atherosclerosis. In contrast, wildtype B cells transferred into μMT^−/−^ ApoE^−/−^ mice increased atherosclerosis and increased CD4 T cells in lesions including activated and memory CD4 T cells. Transferred B cells also increased their expression of atherogenic cytokines IL-1β, TGF-β, MCP-1, M-CSF, and MIF, with partial restoration of germinal centers and plasma immunoglobulins. Our study demonstrates that interaction between B and CD4 T cells utilizing MHCII and CD40 is essential to augment their function to increase atherosclerosis in hyperlipidemic mice. These findings suggest that targeting B cell and CD4 T cell interaction may be a therapeutic strategy to limit atherosclerosis progression.

## Introduction

Crosstalk between B cells and CD4 T cells is required for optimum immune responses in autoimmune diseases, chronic inflammation, transplantation, immunization, and tumors (1–5). It is indispensable for adaptive immune responses where B cells and CD4 T cells confer their effector functions via antibodies and Th1 cytokines respectively (6, 7). In atherosclerosis, immune response following lipid entry into arterial wall is now considered essential for acceleration of established plaques and their subsequent rupture (8). Several lines of evidence show that immune cells are implicated in chronic arterial inflammation. Although macrophages, the first to appear, constitute the majority of cellular infiltrates in all stages of atherosclerotic lesions (9), lymphocytes that accumulate in lesions also contribute to atherosclerosis development and progression depending on their subsets (10).

B cells were once considered atheroprotective due their ability to combat pathogens (11). However, antibody-mediated B cell depletion ameliorated atherosclerosis in both ApoE^−/−^ and LDLR^−/−^ mice suggesting that B cells can be atherogenic (12, 13). These findings are consistent with reports that B cell depletion by anti-CD20 antibody ameliorates autoimmune diseases in humans (14) and in animal models of human diseases (15). Further research identified follicular B2 cells as an atherogenic B cell subset by their capacity to augment atherosclerosis following their adoptive transfer to lymphocyte-deficient and to B cell deficient ApoE^−/−^ mice (13) while B1a cells protect against atherosclerosis by secreting natural IgM antibodies (16). Their derivatives, for example proinflammatory cytokines such as Tumor Necrosis Factor-α (TNF-α) and antibodies such as atherogenic immunoglobulin G (IgG) were shown to be atherogenic (17, 18).

Many CD4 T cell subsets have been implicated in chronic inflammatory diseases (19). CD4 T cells were first reported as atherogenic cells because CD4 T cell transfer into ApoE^−/−^ *scid/scid* mice increased atherosclerosis (20). Later CD28+ CD4+ CD25+ regulatory T cells were found to be a protective CD4 subset in atherosclerosis (21) suggesting that CD28-null CD4 T cells are atherogenic. In chronic inflammation, CD4 T cell can secrete large amounts of Th1 cytokines, TNF-α and IFN-γ (22), that are potent atherogenic cytokines (23). A possible crosstalk between CD4 T cells and B cells in non-lesion areas has been suggested in atherosclerosis (24).

In addition to complete absence of humoral responses in B cell-deficient mice, T cell-responses were also partially impaired and these mice failed to promote inflammatory responses against infection (25, 26). Follicular B cells communicate with follicular helper CD4 T cells utilizing major histocompatibility complex class II (MHCII)-T cell receptor (TCR) and CD40-CD154 interactions and their cognate interactions provides downstream signaling for final development of antigen-specific plasma cells and antigen-specific CD4 T cells (27).

Using B cell-deficient atherogenic ApoE^−/−^ mouse model, we examined atherosclerosis development in the absence of B cells and examined atherosclerosis development again after transfer of different B cells. We found compelling evidence that B cells interact with CD4 T cells using MHCII and CD40 molecules to increase atherosclerosis development.

## Materials and Methods

### Mice and Diet

ApoE-deficient (ApoE^−/−^) and CD40-deficient (CD40^−/−^) mice purchased from the Jackson laboratories, B cell-deficient (μMT^−/−^) mice, a gift from Rajewsky and μMT^−/−^ ApoE^−/−^ mice were maintained at the Precinct animal Centre (PAC), Alfred Research Alliance (ARC), Melbourne Australia. MHCII-deficient (MhcII^−/−^; ${\text{A}}_{\beta }^{-/-}$) mice (28) were from the Melbourne University. All animal experiments approved by the animal ethic committee of ARC were carried out at PAC. Experimental mice (male 6–8 week old) were given ad libitum access to a high fat diet consisting of 21% fat and 0.15% cholesterol (Specialty Feeds, Glen Forrest, Western Australia) and sterile water.

### Genotyping Assessment of μMT and ApoE Deficiencies in μMT^−/−^ ApoE^−/−^ Mice

Tail DNAs, extracted using DNeasy blood and tissue kit (Qiagen, Germany) were subjected to PCR application to examine the genetic deficiency of μMT and ApoE genes. PCR reaction contained 20 mM Tris-HCl pH 8.4, 50 mM KCl, 1.5 mM MgCl_2_, 0.2 mM dNTP, 0.2 μM of each pairs of primer and 0.2 unit of *Taq* DNA polymerase (Invitrogen). PCR condition was as follow: initial denaturation step of 5 min at 95°C, 35 cycles of 10 s at 95°C, 30 s at 60°C and 30 s at 72°C, and final amplification step of 5 min of 72°C. Primers used (Genework, Australia) were as below:

ApoE-Com - 5′-GCC TAG CCG AGG GAG AGC CG-3′,
ApoE-WT - 5′-TGT GAC TTG GGA GCT CTG CAG C-3′,
ApoE-KO - 5′-GCC GCC CCG ACT GCA TCT-3′,
μMT-Com - 5′-CCG TCT AGC TTG AGC TAT TAG G-3′,
μMT-WT - 5′-GAA GAG GAC GAT GAA GGT GG−3′ and,
μMT-KO - 5′-TTG TGC CCA GTC ATA GCC GAA T-3′.

PCR products were separated on 1.5% TAE agarose gel stained with ethidium bromide, visualized using UV light, and digitally recorded.

### Experimental Designs

To determine the interaction between B and CD4 T cells and their effect on atherosclerosis, atherogenic mice with life-long B cell deficiency (μMT^−/−^ ApoE^−/−^) were fed a HFD for 8 weeks and B cell-competent atherogenic mice (ApoE^−/−^) were used as control mice. Next, B2 cells from different donor mice were isolated using magnetic B cell isolation kit (Miltenyi Biotec). Briefly splenocytes were incubated with biotinylated monoclonal anti-CD43, anti-CD4, and anti-Ter119 antibodies, followed by anti-biotin microbeads. Using magnetic columns, B2 cells were negatively purified. As B1cells express CD43, the unlabelled fraction contains only CD19^+^ CD5^−^ B2 cells and it was confirmed by FACS. B2 cell purity was about 98% and viability as assessed by Trypan Blue exclusion was more than 99%. Purified B2 cells were adoptively transferred to μMT^−/−^ ApoE^−/−^ mice (5 × 10^6^ cells via tail vein) at the beginning of 8 week HFD feeding. No cell transfer and wildtype B cell transfer were used as negative and positive controls in comparison to MHCII^−/−^ and CD40^−/−^ transfers. At the end of experiment, mice were killed, collected different tissues to compare B and CD4 T cell effector function and atherosclerosis.

### Tissue Collection at Completion of Experiment

Plasma collected from citrated blood were kept at −80°C for plasma lipid and immunoglobulin determination. Aortic sinus and spleens embedded in OCT media were kept at −80°C for histological, immunohistochemical and immunofluorescent staining. Aortic arches snap-frozen in lipid nitrogen were kept at −80°C to determine plaque cytokine mRNA expression. Immune cells from whole blood, peripheral lymph nodes, spleens, and peritoneal cavities were collected to determine immune cell profile.

### FACS-Assisted Immune Cell Assessment

Single cell suspensions from different tissues were stained with fluorochrome-labeled antibodies (BD-Pharmingen) to identify CD19^+^ B cells and CD4 T cells as previously described (13, 16, 29–31). BD FACSCanto II (BD Biosciences) was used to acquire data and FACSDiva software (BD Biosciences) was used to analyse the data.

### T Cell Proliferation Assay

Splenocytes were labeled using CellTrace Violet (CTV) fluorescent dye (Molecular Probes, Invitrogen) according to manufacturer's instruction. Briefly, 1 × 10^6^ splenocytes were labeled in 1 ml of PBS with 1 μl of CTV (5 mM) at 37°C for 20 min, washed twice with 10% fetal calf serum-containing RPMI 1640 (Invitrogen), and finally resuspended in RPMI 1640/10% FCS. CTV-labeled splenocytes were cultured at a concentration of 0.5 × 10^6^/ml in RPMI 1640/10% FCS in a total volume of 200 μl in 96 well U-bottomed plates (BD Bioscience). Cells stimulated with 2 μg/ml concanavalin A (Sigma) or 2 μg/ml MDA-LDL were cultured at 37°C in 5% CO_2_ for 72 h before staining with PI, anti-CD4 and anti-TCR-b antibodies and analyzed by FACS.

### Determination of Plasma Lipids

Plasma lipid profiles were determined as described before (13).

### Determination of Plasma Total and ox-LDL Immunoglobulins

ELISA was carried to determine plasma total and ox-LDL immunoglobulins as described before (16, 30).

### Determination of Plasma BAFF

Plasma BAFF levels were measured according to manufacturer's instructions using Mouse BAFF/BLyS/ TNFSF13B Immunoassay (R&D systems) as described before (29).

### Histological Staining

Frozen section of aortic sinus embedded in OCT were stained with oil red-O (ORO) to identify the lipid-rich atherosclerosis lesion in vascular intimal areas. Using Optimas 6.2 Video Pro-32 (Bedford Park, South Australia, Australia), total intimal lesion areas and ORO-stained lipid areas were quantified and lipid accumulation areas were corrected using total intimal lesions areas as described before (31).

### Immunohistochemical Staining

Anti-CD68 (Serotec), anti-IgG (BD Pharmingen), and anti-IgM (BD Pharmingen) antibodies were used to assess macrophage accumulation and deposits of immunoglobulins in atherosclerotic lesions as described (30). Anti-CD4 and anti-CD8 antibodies (BD Pharmingen) were used to determine the numbers of CD4 and CD8 T cells in atherosclerotic lesions (31). After staining cell nuclei with Hematoxylin, all sections were visualized under light microscope. All immune cell accumulations were presented per mm^2^.

### Immunofluorescent Staining

Fluorescence-labeled antibodies (BD Pharmingen) were used in immunofluorescent staining of different frozen tissue sections. Anti-CD19 antibody was used to stain B cells in aortic sinus and anti-B220, anti-CD3 or anti-CD4 antibodies were used to stain B and CD4 T cells in spleens. After counterstaining with either DAPI or Hoechst 33342, the sections were visualized under fluorescence microscope. In some experiments, PD-1 and Bcl-6 antibodies together CD4 antibody were used to identify CD4 T follicular helper cells.

### Determination of mRNA Expression

Total RNA from aortic arches were extracted using RNeasy Fibrous Tissue Mini Kit (Qiagen) as per manufacturer's instruction. After determining RNA concertation, 10 ng of total RNA was used in OneStep QuantiFast SYBR Green RT-PCR (Qiagen) with appropriate housekeeping genes and controls as described before (16, 29, 31). Primer used are as follow:

IL1-β sense (S) - 5′-CCA CCT CAA TGG ACA GAA TCT CAA-3′,
IL1-β antisense (AS) - 5′-GTC GTT GCT TGG TTC TCC TTG T-3′;
TGF-β (S) - 5′-AGC CCT GGA TAC CAA CTA TTG C-30;
TGF-β (AS) - 5′-TCC AAC CCA GGT CCT TCC TAA-30,
MCP-1 (S) - 5′-CTC AGC CAG ATG CAG TTA ACG-3′,
MCP-1 (AS) - 5′-GGG TCA ACT TCA CAT TCA AAG G-3′;
M-CSF (S) - 5′-GGA GTA TTG CCA AGG AGG T-3′,
M-CSF (AS) - 5′-GAC TGT CGA TCA ACT GCT-3′;
MIF (S) - 5′-GGC AAG CCC GCA CAG TAC-3′,
MIF (AS) - 5′-ATC GTT CGT GCC GCT AAA AGT-3′;
TNF-α (S) - 5′-TCT TCT GTC TAC TGA ACT TCG-3′,
TNF-α (AS) - 5′-GAA GAT GAT CTG AGT GTG AGG-3′;
IFN-γ (S) - 5′-CTG GAC CTG TGG GTT GTT GAC-3′,
IFN-γ (AS) - 5′-CAA CAG CAA GGC GAA AAA GG-3′;
IL-12 (S) - 5′-GGT GTA ACC AGA AAG GTG CG-3′,
IL-12 (AS) - 5′-GAG GAA TTG TAA TAG CGA TCC TGA G-3′;
IL-17 (S) - 5′-TTC ATC TGT GTC TCT AGT GCT-3′,
IL-17 (AS) - 5′-AAC GGT TGA GGT AGT CTG AG-3′.

### Statistical Analysis

Kolmogorov-Smirnov test was used to assess normal distribution of the data. Unpaired two sided student-*t* test or Mann-Whitney *U*-test, depending on normal distribution was used to calculate p value. Statistically significance was considered if *p* < 0.05. Data were expressed as mean ± SEM.

## Results

### Life-Long Deficiency of B Cells Reduces Atherosclerosis

C57Bl/6 mouse deficient in ApoE protein (ApoE^−/−^) and C57Bl/6 mouse deficient in B cells by targeted disruption of the immunoglobulin μ-heavy chain gene (μMT^−/−^) were crossed to generate a permanent global B cell deficiency in ApoE^−/−^ mice (μMT^−/−^ApoE^−/−^). Genotypes of μMT^−/−^ ApoE^−/−^ mice were confirmed by polymerase chain reaction-gel electrophoresis (Figures S1A,B). B cells in μMT^−/−^ApoE^−/−^ mice assessed by FACS analysis confirmed complete absence of B cells in peripheral blood, lymph nodes, spleens and peritoneal cavities (Figure S1C).

**Figure 1:**
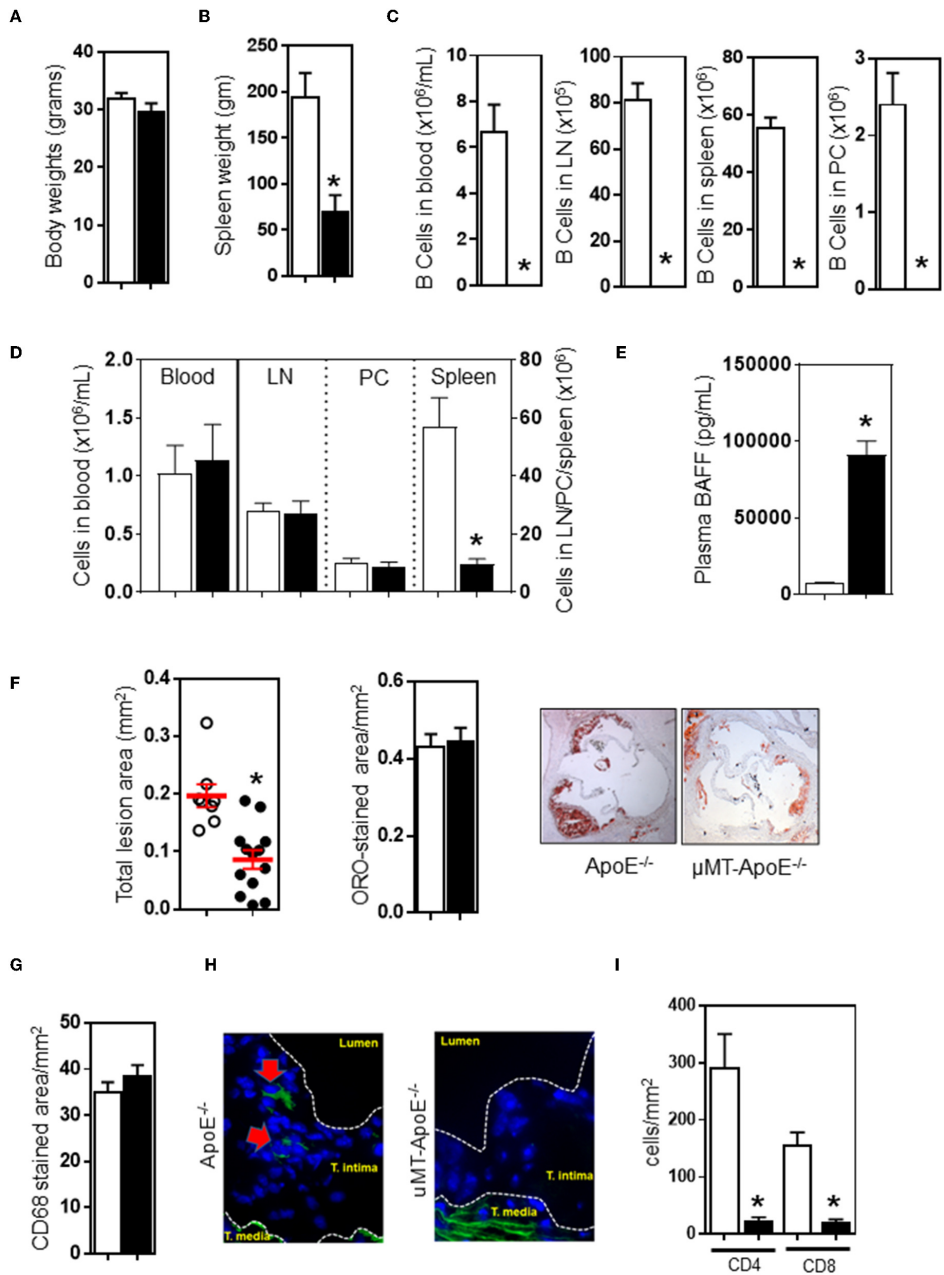
B cell deficiency reduced atherosclerosis, abolished B cells and reducedCD4 T cells in atherosclerotic lesions. ApoE^−/−^ and μMT^−/−^ ApoE^−/−^ mice (male 6–8 week-old) were fed a high fat diet for 8 weeks. At the end of experiment, B cell deficiency did not affect **(A)** body weight, **(B)** reduced spleen weight, and **(C)** completely abolished B cells in different tissues. CD4 T cells were unaffected **(D)** in peripheral blood, lymph nodes, and peritoneal cavities however spleen T cells were severely reduced. **(E)** B cell deficiency in μMT^−/−^ ApoE^−/−^ mice was associated with augmented plasma BAFF levels. B cell-deficient μMT^−/−^ ApoE^−/−^ mice showed reduced atherosclerosis as assessed by **(F)** total intimal lesion area and Oil Red-O stained lipid accumulation, however lipid content corrected as per lesion size was not affected. **(G)** Macrophage accumulation as corrected to lesion was not affected, but **(H)** B cells were completely absent and **(I)** CD4 T cells were reduced in atherosclerotic lesion of B cell-deficient mice. Representative microimages and histograms shown. Data presented as mean ± SEM of two to three independent experiments. *n* = 12–15 per group, *p < 0.05, □ ApoE^−/−^ mice ■ μMT^−/−^ ApoE^−/−^ mice.

To investigate the effect of a congenital B cell deficiency on atherosclerosis development, atherosclerosis was generated by feeding ApoE^−/−^ mice a high fat diet (HFD) for 8 weeks. Genetically targeted-deletion of the immunoglobulin μ-heavy chain gene in μMT^−/−^ApoE^−/−^ mice did not affect body weight (Figure 1A), but reduced spleen weight by ~65% (Figure 1B). In agreement with phenotype results of μMT^−/−^ApoE^−/−^ mice (Figures S1A–C), FACS analysis at the end of experiment showed that no B cells were detected in the peripheral blood, lymph nodes, spleens and peritoneal cavities in μMT^−/−^ApoE^−/−^ mice (Figure 1C). CD4 T cells in peripheral blood, lymph nodes and peritoneal cavities were unaffected albeit with smaller lymph nodes, but their numbers in spleen were reduced by ~85% when B cells were genetically depleted (Figure 1D). Complete loss of B cells in μMT^−/−^ApoE^−/−^ mice resulted in increased plasma BAFF levels that by multiple folds compared to B cell-sufficient ApoE^−/−^ mice, suggesting attempted homeostatic compensation (Figure 1E). Despite having comparable body weights (Figure 1A) and plasma lipids (Figure S2), lifelong deficiency of B cells reduced atherosclerosis. Compared to ApoE^−/−^ mice, atherosclerosis at the aortic sinus in μMT^−/−^ApoE^−/−^ mice was reduced by ~50% as assessed by intimal atherosclerotic lesion areas, without affecting lipid composition in the lesions (Figure 1F).

Next, we investigated the lesion content of macrophages and lymphocytes accumulated in atherosclerotic lesions as they contribute to atherosclerosis development and progression (9, 10). Immunohistochemical analysis of atherosclerotic lesions at the aortic sinus revealed that composition of CD68^+^ macrophage was not affected by B cell deficiency (Figure 1G). As expected, immunofluorescent staining showed that B cells were undetected in atherosclerotic lesions of μMT^−/−^ ApoE^−/−^ mice whilst B cell-competent ApoE^−/−^ mice showed presence of CD19^+^ B cells in atherosclerotic lesions (Figure 1H). We also assessed T cells in atherosclerotic lesions and found that less CD4^+^ T cells (ApoE^−/−^ vs. μMT^−/−^ApoE^−/−^ mice: 292 ± 59 vs. 24 ± 6 cells/mm^2^) and CD8 T cells (ApoE^−/−^ vs. μMT^−/−^ApoE^−/−^ mice: 158 ± 20 vs. 21 ± 5 cells/mm^2^) were recruited into lesions when B cells were deficient (Figure 1I).

### Reduced Atherosclerosis in B-cell Deficient Mice Is Associated With Reduced Immunoglobulin Levels

B cells interact with follicular CD4 T cells to initiate their activation and proliferation leading to terminal differentiation of immunoglobulin-producing plasma cells (27, 32). B and T cell interaction in turn initiates activation of follicular CD4 T cells and their effector function (27). Therefore, we first asked whether lifelong B cell deficiency affects B and T cell interaction in spleens. As expected, spleen B cells were completely absent in B cell-deficient mice in contrast to the finding that B cell-competent ApoE^−/−^ mice showed B cells in close proximity to T cells (Figures S3A,B) indicating possible cognate interaction between T and B cells. As B and T cell interaction is required for plasma cell differentiation and production of high affinity immunoglobulins that play a critical role in humoral response, we next assessed the effect of genetic deletion of immunoglobulin μ-heavy chain gene on B cell-derived immunoglobulins. ELISA analysis showed complete absence of total immunoglobulins (Igs), immunoglobulin G (IgG), and immunoglobulin M (IgM) in μMT^−/−^ApoE^−/−^ mice (Figure S3C). Determination of plasma levels of total and ox-LDL-specific immunoglobulins showed that ox-LDL specific immunoglobulins (total, IgG, and IgM) in plasma were detected in B cell-competent ApoE^−/−^ mice, but B cell-deficient μMT^−/−^ApoE^−/−^ mice failed to show presence of these immunoglobulins (Figure S3D). In agreement with the literature (33), B cells and their Ig products were absent in μMT^−/−^ ApoE^−/−^ mice. Further analysis by immunohistochemical staining also showed that no detectable IgG and IgM deposits were observed in atherosclerotic lesions of μMT^−/−^ApoE^−/−^ mice in contrast to abundant immunoglobulin lesion deposits in ApoE^−/−^ mice (Figure S3E).

### B Cell Deficiency Reduces Lesion CD4 T Cells and Proinflammatory Cytokines, IL-1β, TGF-β, MCP-1, M-CSF, and MIF

**Figure 2:**
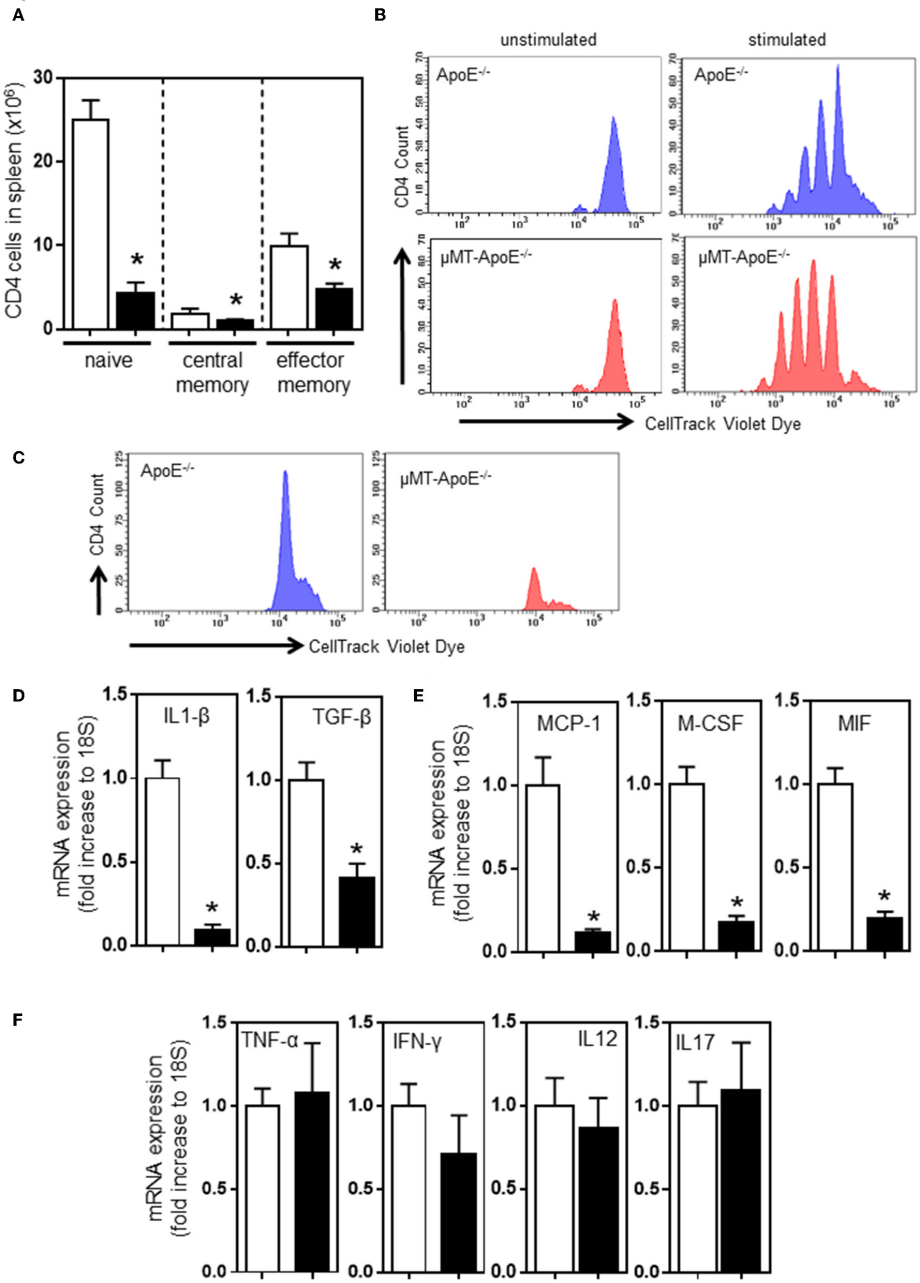
B cell deficiency affects CD4 T cell activation in spleens and inflammation in atherosclerotic lesions. FACS analysis done at the completion of 8 week high fat diet feeding showed **(A)** reduced numbers of naïve (CD44^−^ CD62L^+^), central memory (CD44^+^ CD62L^+^) and effector memory (CD44^+^ CD62L^−^) CD4 T cells in spleens in μMT^−/−^ ApoE^−/−^ mice. Using CellTracking Violet dye, dye-labeled splenocytes (0.5 × 10^6^/ml) were cultured with Concanavalin A (2 μg/ml) or MDA-LDL (20 μg/ml) in 96-well U-bottomed plates (see text for detail). FACS analysis showed CD4 T cell proliferation upon **(B)** systemic non-specific stimulation and **(C)** MDA-LDL specific stimulation. RNAs extracted from atherosclerotic arches of ApoE^−/−^ and μMT^−/−^ ApoE^−/−^ mice were used to determine **(D–F)** mRNA expression of inflammatory cytokines using RT-PCR. Data presented as mean ± SEM of two to three independent experiments. *n* = 12–15 per group, *p < 0.05, □ ApoE^−/−^ mice ■ μMT^−/−^ ApoE^−/−^ mice.

B and T cell interaction results in bi-directional effects. Antigen-experienced activated B cells and memory B cells are responsible for proliferation and differentiation of CD4 T cells (34). Thus, we determined the profile of differential CD4 T cell subsets in spleens. FACS analysis indicated that CD44- CD62L+ naïve, CD44+ CD62L+ central memory, and CD44+ CD62L- effector memory CD4 T cells were reduced in mice (Figure 2A) when B cells were deficient (Figure 1C); however, the reduced numbers of CD4 T cell subset might be due to a smaller spleen size (Figure 1B) and reduced numbers of CD4 T cells in spleen (Figure 1D). CD4 T cells do not require B cells to initiate systemic T cell responses in a B cell-deficient environment (35). We asked if CD4 T cells in μMT^−/−^ ApoE^−/−^ mice can respond and proliferate upon stimulation with concanavalin A and MDA-LDL. CellTrace Violet dye-stained spleen cells stimulated with Concanavalin A for 72 h and then subjected to FACS analysis showed that CD4 T cells from μMT^−/−^ ApoE^−/−^ mice proliferated upon Concanavalin A stimulation (Figure 2B), however CD4 T cell responses to MDA-LDL were reduced in μMT^−/−^ ApoE^−/−^ mice compared to ApoE^−/−^ mice (Figure 2C). This indicates that T cells in μMT^−/−^ ApoE^−/−^ mice are still efficient in their responses to systemic stimuli, yet they fail to respond to antigen-specific stimulation. Indeed, T cells from B cell-deficient environment had poor proliferation to oxLDL-specific stimulation (36).

Our results suggest that congenital B cell deficiency reduces atherosclerosis and recruits less T cells into lesions suggesting the possibility of reduced arterial inflammation. To test this hypothesis, we carried out mRNA expression of cytokines that are crucially required for inflammation and immune cell recruitment. RT-PCR analysis using atherosclerotic aortic arches showed reduced expression of proinflammatory cytokines, IL-1β, TGF-β, MCP-1, M-CSF, and MIF, confirming reduced arterial inflammation (Figures 2D,E). However, expression of TNF-α, IFN-γ, IL-12, and IL-17 were unaffected (Figure 2F). Despite CD4 T cells being able to respond to systemic stimulation, collectively congenital B cell deficiency reduced atherosclerosis by reduced B and T cell effector functions.

### B Cell-Expressed MHCII and CD40 Molecules Are Required for B and T Cell Interaction in Atherosclerosis

**Figure 3:**
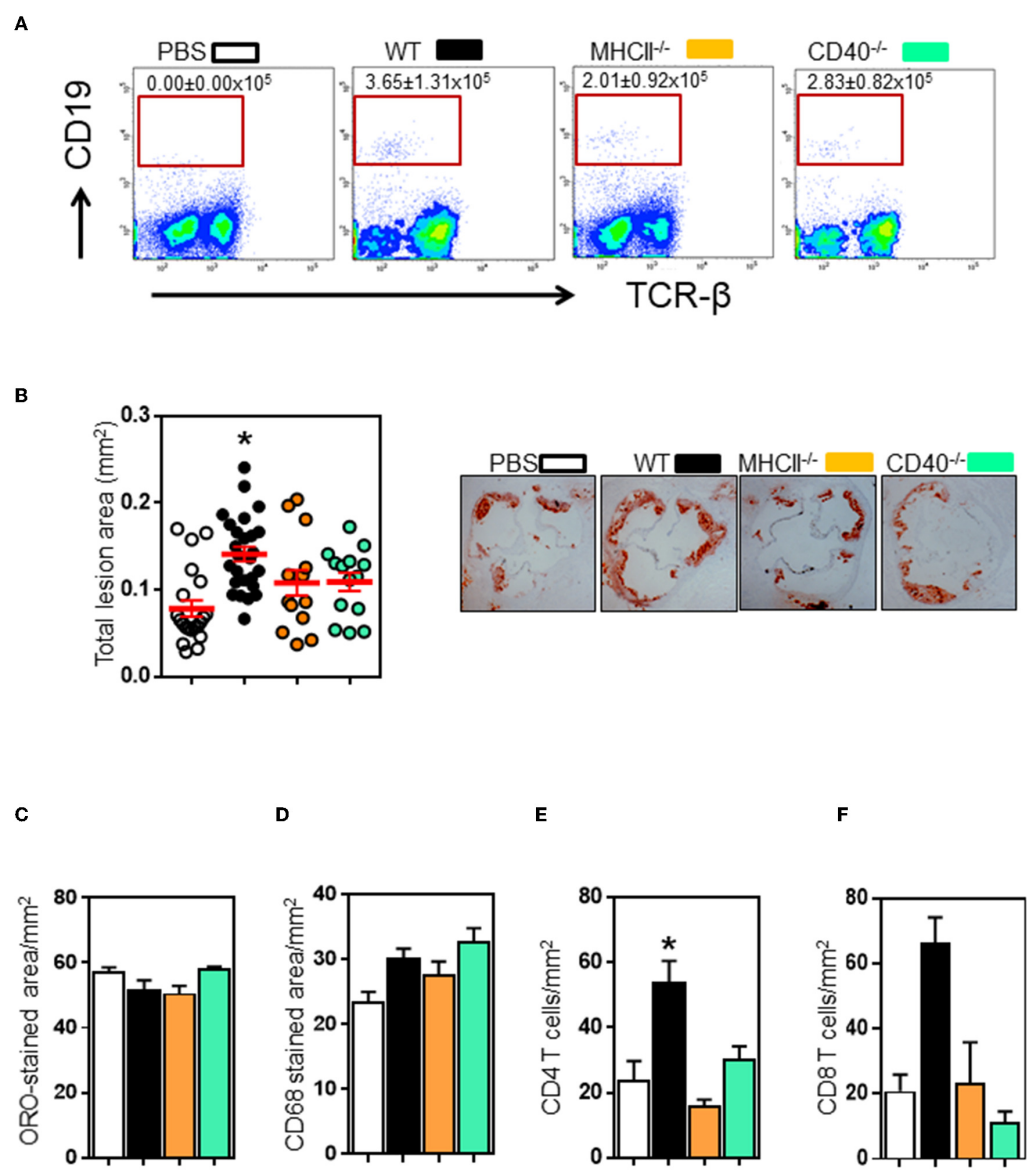
Transfer of wildtype, not MHCII- or CD40-knockout B2 cells promote atherosclerosis in μMT^−/−^ ApoE^−/−^ mice. Spleen B2 B cells isolated and purified from different donors (see text for detail) were adoptively transferred into μMT^−/−^ ApoE^−/−^ mice at the beginning of 8 week HFD. At the end of experiment, FACS analysis showed that **(A)** transferred B cells were detected in recipient spleens. Atherosclerosis at aortic sinus was assessed by **(B)** total intimal lesion areas in ORO-stained aortic sinus sections. Wildtype, not MHCII- or CD40-deficient B2 B cells augmented atherosclerosis without affecting **(C)** lipid and **(D)** macrophage accumulation expressed as per lesion area. Lesion immune cell analysis showed that **(E–F)** CD4 T and CD8 T cell accumulation was reduced in μMT^−/−^ ApoE^−/−^ mice. Data presented as mean ± SEM of two independent experiments. *n* = 9 or more per group, *p < 0.05 compared to PBS transferred group. □ PBS transfer, ■ WT B cell transfer, 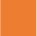 MHCII^−/−^ B cell transfer, and 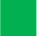 CD40^−/−^ B cell transfer.

Follicular B cells communicate with CD4 follicular T cells through MHCII and CD40 molecules (34). To determine the role of these molecules in B and T cell interaction in atherosclerosis, we adoptively transferred spleen B cells isolated from different donors (Figure S4) into B cell-deficient μMT^−/−^ApoE^−/−^ mice (5 million B220+ B cells, i.v tail vein) at the beginning of 8 weeks HFD. FACS analysis showed transferred B2 cells in recipient spleens at the end of 8 weeks HFD (Figure 3A). Wildtype B cell recipients had more B cells, yet statistically not significant. Atherosclerosis in μMT^−/−^ ApoE^−/−^ mice that received wildtype B2 B cells increased by 109% as assessed by intimal lesion area (Figure 3B). In contrast, despite having B cells detected (Figure 3A), comparable body weights (data not shown) and plasma lipids (Figure S5), while atherosclerosis was an increasing trend with mice that received MHCII^−/−^ and CD40^−/−^ B cells, it did not reach statistical significance (Figure 3B). Lipid and macrophage accumulation corrected for lesion area was unaffected (Figures 3C,D). As we have shown that lifelong B cell deficiency reduces lesion CD4 and CD8 T cell recruitment (Figure 1I), we asked if their accumulation in atherosclerotic lesions in μMT^−/−^ ApoE^−/−^ mice were increased after transfer of B2 cells. We found that CD4 and CD8 T cell accumulations were increased by 2 to 3-fold in atherosclerotic lesions following transfer of wild-type B2 cells (Figures 3E,F). In contrast CD4 and CD8 T cells were not increased in mice that received B cells deficient in either MHCII or CD40 (Figures 3E,F).

**Figure 4:**
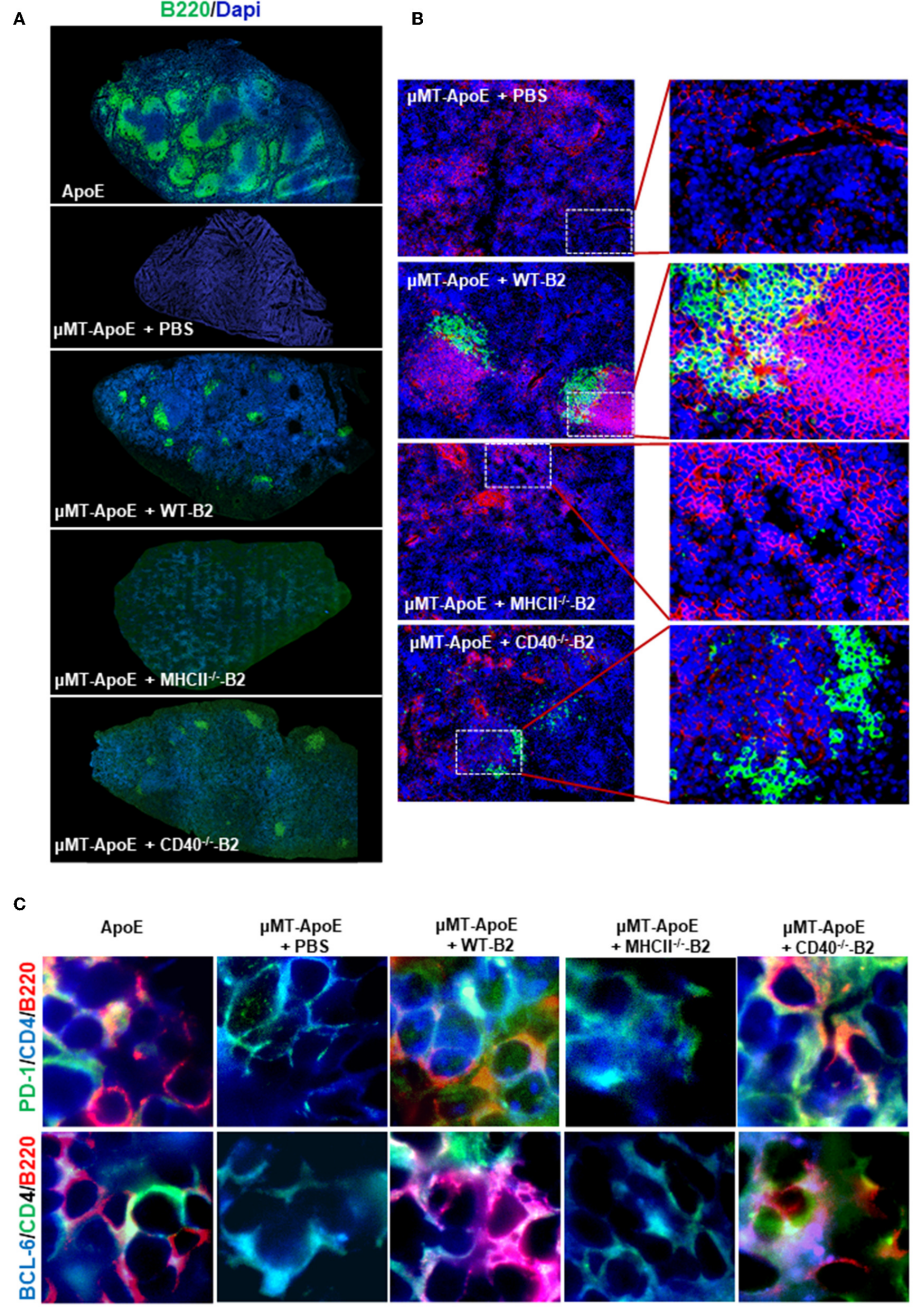
Transfer of wild type and CD40-deficident B2 cells restore B and T cell localization in B cell deficient μMT^−/−^ ApoE^−/−^ mice. In B cell transfer experiments, B2 B cells were adoptively transferred into μMT^−/−^ ApoE^−/−^ mice and spleens were collected at the end of 8 week HFD. Frozen-section of OCT-embedded spleens were sectioned and immunologically stained with various antibodies. Immune fluorescence staining showed **(A)** reappearance of B cell follicles and **(B)** B cell co-localization with T cells in μMT^−/−^ ApoE^−/−^ spleens that received WT or CD40-deficient B cells. **(C)** CD4 T follicular helper cells identified by PD-1 and Bcl-6 in close proximity to spleen B220 B cells, as seen in ApoE^−/−^ mice were observed following WT or CD40-deficient B cell transfer into μMT^−/−^ ApoE^−/−^ mice, however neither PBS nor MHCII-deficient B cell transfer produced such observation. Representative photomicrographs from three different experiments.

Antigen-experienced B cells migrate to T cell-rich regions to interact with antigen-specific CD4 T cells to initiate their terminal differentiation to plasma cells as well as to facilitate CD4 T cell effector function. To investigate if transferred B cells can interact with CD4 T cells in secondary lymphoid organs, we examined whether B cells are in close proximity to T cells in spleens. Immunofluorescence staining showed appearance of well-organized B cell follicles and co-localization between B and T cells in mice that received wildtype or CD40-deficient B cells compared to B cell-deficient μMT^−/−^ApoE^−/−^ mice (Figures 4A,B). However, B cells deficient in MHCII failed to generate well-organized B cell follicles nor co-localized with T cells despite their presence in spleen confirmed by Immunofluorescence staining (Figure 4A) and FACS (Figure 3A). The data supports the essential role of MHCII-mediated antigen presentation between antigen-experienced B and CD4 T cells. Using multiple antibodies in immunofluorescence staining, we identified CD4 follicular helper T (Tfh) cells as identified by either PD-1 or Bcl6 co-expression in close proximity to spleen B220 B cells (Figure 4C). These cell-to-cell interactions between CD4 Tfh cells and B cells were only observed in mice that received wildtype and CD40-deficient B2 cells, not in those that received MHCII-deficient B2 cells (Figure 4C), suggesting the essential requirement of MHCII molecule compared to CD40 molecule in their interactions.

### B Cell-Specific MHC II and CD40 Expressions Are Required for Both B and CD4 T Cell Effector Functions

**Figure 5:**
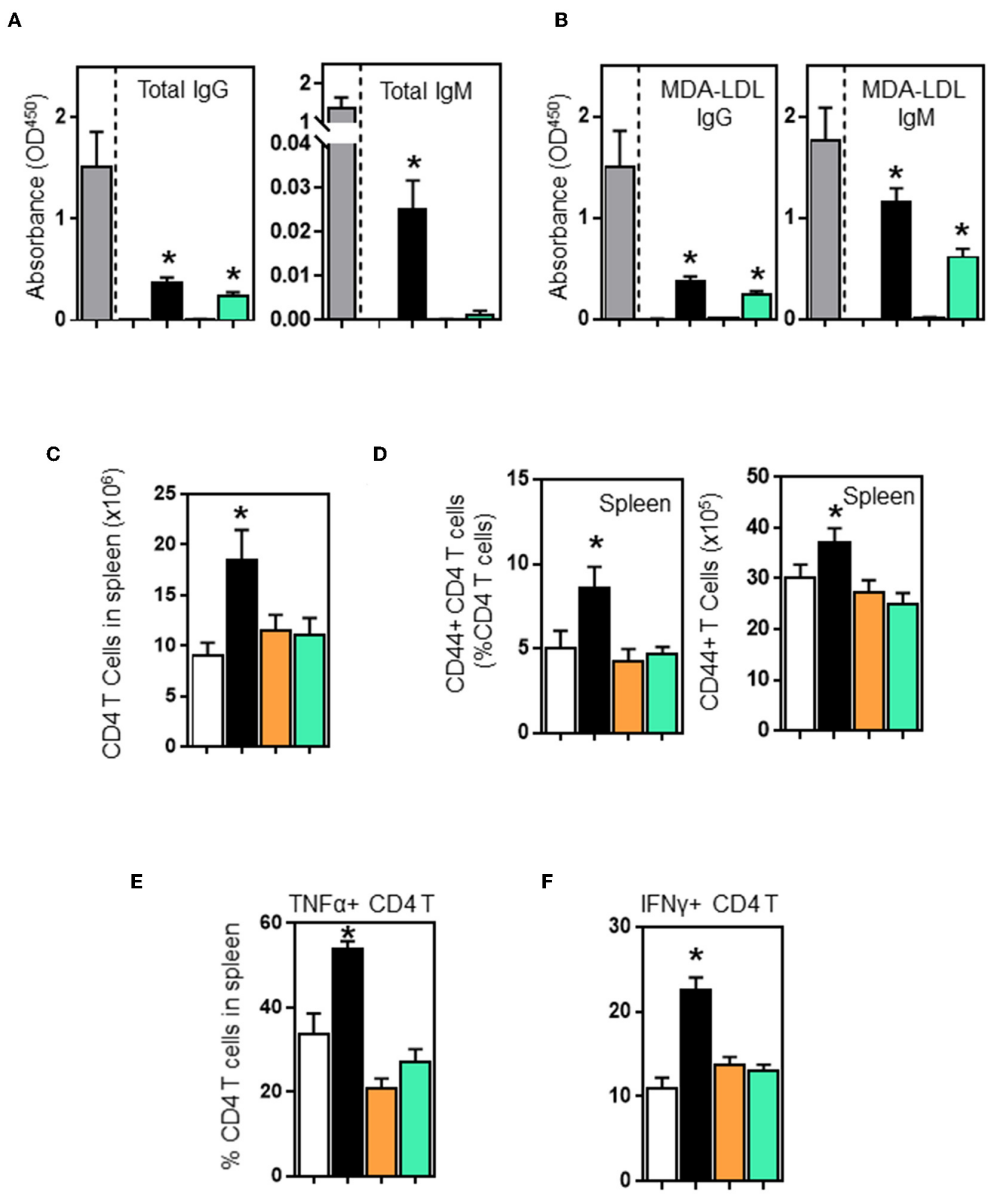
Wildtype and CD40-deficient B2 cells restore plasma immunoglobulins, however only wildtype B2 transfer enhances CD4 T cell activation and their production of TNF-α and IFN-γ in μMT^−/−^ ApoE^−/−^ mice. B2 B cells were adoptively transferred into μMT^−/−^ ApoE^−/−^ mice at the beginning of 8 week HFD. At the end of experiment, plasma immunoglobulin levels determined by ELISA showed that wild-type and CD40^−/−^ B2 B cell transfer groups increased **(A)** total IgG, total IgM and **(B)** oxLDL-IgG and oxLDL-IgM subclasses. However, only wildtype B2 cells increased **(C)** total CD4 T cells, **(D)** activated CD44^+^ CD4 T cells and **(E)** TNF-α- and **(F)** IFN-γ-producing CD4 T cells in spleens. Data presented as mean ± SEM of two to three independent experiments. *n* = 9 per group, *p < 0.05 compared to PBS transferred group. □ PBS transfer, ■ WT B cell transfer, 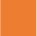 MHCII^−/−^ B cell transfer, and 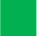 CD40^−/−^ B cell transfer.

B and T cell interactions affect effector functions of both B and T cells. B cell deficiency in μMT-ApoE^−/−^ mice not only completely abolished total immunoglobulin production but also significantly reduced activated CD4 T cells. To investigate whether adoptive transfer of B cells into μMT^−/−^ ApoE^−/−^ mice restores these effector functions, we first assessed plasma immunoglobulin levels in μMT^−/−^ ApoE^−/−^ mice that received B2 cells. ELISA determination revealed that plasma total IgG levels in wild-type and CD40-deficient B2 cell transfer groups were increased by 23 and 18%, respectively compared to the total IgG levels of ApoE^−/−^ mice. However, total IgM levels in WT and CD40-deficient B2 cell transfer groups were detectable at low levels compared to ApoE^−/−^ mice. In contrast, total IgM were not detected in PBS and MHCII-deficient B2 cell transfer groups. When comparing with PBS transfer group, total IgM levels in WT B cell transfer group was increased, however CD40-deficient B2 cell transfer groups had increased total IgM levels that failed to reach statistical significance (*p* = 0.0757) (Figure 5A). IgG and IgM antibodies specific to oxLDL in μMT^−/−^ ApoE^−/−^ mice that received wild-type B2 cells were increased 25 and 66%, respectively whilst mice that received CD40-deficient B cells increased oxLDL-specific IgG and IgM levels by 16 and 35%, respectively compared to those from ApoE^−/−^ mice (Figure 5B). But mice that received either PBS or MHCII-deficient B cells failed to increase plasma total or MDA-LDL-specific immunoglobulin levels (Figures 5A,B). Collectively our data suggest an indispensable role of MHCII in humoral response in agreement with the finding that IgG and IgM antibodies are detected in CD40-deficient mice (37). Next, we determined the number of CD4 T cells in spleens; FACS analysis indicated that more CD4 T cells were detected in mice that received wildtype B cells compared to PBS-control group (Figure 5C). In contrast, mice that received B cells deficient in either MHCII or CD40 showed an increasing trend in the number of spleen CD4 T cells, but did not reach statistical significance (Figure 5C). Further analysis showed that with the wildtype B cell transfer, there was not only an increase in number of activated CD4 T cells (Figure 5D) but there was also an increase in number of TNF-α and IFN-γ producing CD4 T cells (Figures 5E,F). B cells deficient in either MHCII or CD40 failed to activate CD4 T cells nor increase CD4 T cells expressing TNF-α and IFN-γ (Figures 5E,F), highlighting the critical role of MHCII and CD40 expressing on B cells in Th1 l responses accordance with literature (27, 38) and in agreement with a significant reduction in spleen IFN-γ+ CD4+ T cells in B cell-depleted mice (12).

## Discussion

Several lines of evidence support our conclusion that B cells in concert with CD4 T cells promote atherosclerosis. B and CD4 T cells are recognized for their contribution to inflammation (39, 40). Chronic depletion of CD4 T cells by anti-CD4 antibody in MRL/*lpr* mice reduced inflammation, arthritis, nephritis, and dsDNA antibodies (41). When B cells were congenitally deficient, HFD-fed obese mice did not develop type 2 diabetes, their fasting sugar, and insulin tolerance tests were unaffected, but helper CD4 T cell function was ameliorated (42). These studies suggest interactions between B and CD4 T cells that are required for inflammation progression.

Humoral and cellular immune responses are implicated in development and progression of atherosclerosis. Whilst IgM antibodies are atheroprotective, the role of IgG antibodies in atherosclerosis remains uncertain (43, 44). Chimeric atherogenic mice selectively deficient in transcription factor XBP1 on B cells (irradiated LDLR-deficient mice, transplanted with Cd79a^Cre/+^ Xbp1^fl/fl^ bone marrow cells) had reduced serum IgG level, and reduced atherosclerosis (45). However, as these mice also displayed reduced IgM levels, it is not clear whether the reduced atherosclerosis is due to IgM loss in chimeric mice. A recent study also showed that absence of serum IgG and IgM reduced atherosclerosis in Prdm1^fl/fl^ Cd19^cre/+^ ApoE^−/−^ mice (46), suggesting pathogenic properties of IgGs in atherosclerosis progression. We have also reported that follicular B cells promote atherosclerosis by T cell-mediated differentiation into plasma cells and secreting pathogenic IgG (18). Transfer of IgG purified from hyperlipidemic ApoE^−/−^ mice increased atherosclerosis in contrast to transfer of IgG purified from normolipidemic mice, consistent with an atherogenic role of IgG in atherosclerosis (18).

In addition to pathogenic antibodies that interact with lesion constituents, B cells can confer their atherogenicity locally. We have shown that B cell-derived TNF-α augments macrophage production of TNF-α in atherosclerosis (17). Our data indicate that B deficiency in atherogenic mice reduces inflammation and atherosclerosis by two distinct pathways. Firstly, B cell deficiency has the direct effect on reducing atherosclerosis via pathogenic antibodies and cytokines. Secondly, B cell deficiency impairs CD4 T cell pathogenicity in atherosclerosis. Furthermore, there may be additional mechanisms through increased plasma BAFF levels, as a result of B cell deficiency. BAFF, a critical survival factor for B cells, has anti-inflammatory effects as BAFF overexpression or neutralization reduced atherosclerosis (47, 48). In addition to BAFF Receptor (BAFFR), BAFF also interacts with B cell maturation antigen (BCMA), and TNFR homolog transmembrane activator and Ca^2+^ modulator and CAML interactor (TACI). The anti-inflammatory action of BAFF is mediated by their interaction with either B1a cells or macrophages via BAFF-TACI interaction (47, 48).

CD4 T cells confer their pathogenicity by two pathways in autoimmune diseases and chronic inflammation. Firstly, CD4 helper T cells migrate to germinal centers in secondary lymphoid centers to produce cytokines such as IL-21 and IL-4 that promote B cell differentiation into plasma cells in germinal centers (49, 50). The finding is consistent with the report that Tfh cell deficiency impair germinal center formation and plasma cell differentiation (51, 52). Secondly, CD4 T cell interaction with follicular B cells promotes CD4 T cell activation and differentiation to effector and memory CD4 T cells (50). Our finding that TNF-α and IFN-γ producing CD4 T cells in spleens increased following WT B cell transfer into μMT^−/−^ ApoE^−/−^ mice is consistent with their migration to chronically inflamed arterial plaques. This is further supported by reduced CD4 T cells when B cells are absent. Both proinflammatory TNF-α and IFN-γ cytokines are atherogenic. We and others have reported that B cell depletion reduced CD4 T cell activities (12, 13, 30, 53). The lack of specificity of current CD4 T cell depletion strategies prevented us from directly assessing the importance of the reduction in CD4 T cell activity on atherosclerosis. T cell depletion strategies depleted both CD4 and CD8 T cells; the latter also being important in atherosclerosis (31) and antibodies depleting CD4 T cells also deplete CD4+ NKT cells which also contribute to atherosclerosis (54).

Our data from depletion and transfer experiments suggest that B cells play a role in T cell trafficking to local inflammatory sites. CD4 T cells, reduced in atherosclerotic lesions of μMT^−/−^ ApoE^−/−^ mice increased to double their numbers following WT B2 cell transfer. Immune cell migration depends on MCP-1, MIF and M-CSF. Genetic deficiencies of MCP-1, MIF, and M-CSF reduced immune cell accumulation in atherosclerotic lesions (55–57). MCP-1 regulates migration and infiltration of monocytes, memory T lymphocytes, and other immune cells (58). The observation suggests that MCP-1 is required for recruiting immune cells, resulting in augmented immune responses in atherosclerotic lesions. A critical role of CD40-CD154 interaction for CD4 T cell migration has been demonstrated in viral and experimental autoimmune encephalitis (59, 60). In the absence of B cells, CD4 T cell accumulation is reduced in insulitis (61). Pro inflammatory cytokine IL1β produced by activated macrophages modulates immune responses and apoptosis. Genetic deficiency of IL-1 receptor 1 or treatment with anti-IL1β decreased atherosclerosis in the aortic sinus and total aorta of ApoE^−/−^ mice (62, 63). The finding of reduced macrophage accumulation in atherosclerotic lesions and less arterial expression of IL1β in μMT^−/−^ ApoE^−/−^ mice is consistent with the reduced inflammation in atherosclerotic lesion, and decreased atherosclerosis in μMT^−/−^ ApoE^−/−^ mice. Collectively our results demonstrate key roles provided by B2 cells in recruiting CD4 T cells to arterial lesions in atherosclerosis development.

Our study has also shown that congenital B cell deficiency in μMT^−/−^ ApoE^−/−^ mice decreases atherosclerosis by down-regulating macrophage attractant chemokines, reducing total lesion macrophage numbers, and decreasing inflammation in atherosclerotic lesions. Our finding of reduced atherosclerosis in μMT^−/−^ ApoE^−/−^ mice is consistent with recent reports of B2 cells as proatherogenic B cells in atherosclerosis (12, 13, 29, 30, 53). It is also consistent with human and murine studies where B cells also promote their pathogenicity by antibody-unrelated mechanisms because anti-CD20 targeted B cell depletion ameliorates autoimmune diseases without affecting levels of plasma autoantibodies (64, 65). Our study contributes to a better understanding of B cell pathogenicity in atherosclerosis and provides insights for development of B-cell targeted therapies.

B cells act as antigen presenting cells to induce T cell responses to specific antigens. B cells also have roles in development and maintenance of memory T cells (6, 66–68) to provide optimal T cell response to antigens in long-term antigen exposure. However, systemic T cells responses are observed in a B cell-deficient environment (35, 69, 70) suggesting a role for B cells in CD4 T cell responses to specific antigens. We have shown that CD4 T cells from μMT^−/−^ ApoE^−/−^ mice respond and proliferate upon Concanavalin A stimulation. We propose that while B cells are not critical for activation and induction of T cells in systemic responses, B cells are critically required for CD4 T cells in specific antigen responses, such as lipid antigen in the setting of persistent hyperlipidemia. Indeed, CD4 T cells failed to respond to lipid antigen in B cell-deficient mice (36).

Antigen presenting cells utilize MHCII to present antigens to T cell receptors. Whilst interaction between CD80/CD86 and CD28 is required for Th2 responses (71), linkage between CD40 and CD40 ligand is crucial for activation of Th1 cells and their responses (7). Failure to promote atherosclerosis in MHCII- or CD40-deficient B2 transfer indicates that B2 cells promote atherosclerosis by activating Th1 cells and augmenting Th1 responses. Our observation is in accordance with reports where anti-CD40 antibody treatment reduced atherosclerosis in LDLR^−/−^ mice (72) and inhibition of Th1 responses reduced atherosclerosis in ApoE^−/−^ mice (73). Recently, Wigren et al., and Williams et al., reported that global or B cell-specific deficiency of MHCII were associated with low level of total IgG and IgM as well as low or undetectable oxLDL specific antibodies (74, 75), supporting our data by defining differential roles of a B cell-specific MHCII and CD40 in generation of plasma cells and antibodies. Interestingly, in contrast to increased atherosclerosis in MHCII^−/−^ ApoE^−/−^ double knockout mice (74), MHCII deficiency on B cells did not affect atherosclerosis in LDLR^−/−^ mice despite large but incomplete reductions in MHCII in B cells (75); reductions resulted in reduced IgG1 and IgG2c but not IgG2b nor IgM. Incomplete depletion of MHCII from B cells is known to lead to strong selection of escaped B cells where MHCII is not deleted in the activated and plasmablast compartments (76, 77), which may partially explain the latter observation. Specific autoantibody IgG subtypes differ in their dependence on B cells MHCII expression (76). Our data showing detectable, but very low level of MDA-LDL specific antibodies in B cell transfer experiments proposes that a sensitive ELISA is critically essential for detecting low-level antibodies.

Our finding that atherosclerosis is reduced following B cell deficiency arising from genetic deficiency of the Ig heavy chain in μMT^−/−^ApoE^−/−^ mice is at variance with a previous report that atherosclerosis is increased in LDLR^−/−^ mice rendered chimeric for μMT by lethal irradiation and bone marrow transplantation (36) to induce B cell deficiency. However, irradiation not only alters the pattern of lesions but also the characteristics of developing lesions (78). Atherosclerotic lesions in mice subjected to bone marrow transplantation contain more lipid, larger lipid cores and greater macrophage numbers than do lesions of mice not subjected to bone marrow transplantation (78). Bone marrow transplantation alone does not necessarily fully reconstitute the immune system (79). For example, following bone marrow transplantation reconstitution of γδ T cells is relatively poor (79) and this may enhance αβ T cell activity (80). Whether such mechanisms explain the different results of B cell depletion following genetic depletion of B cells and transplantation of bone marrow deficient in B cells remains to be determined. Similar discrepancies between genetic deletion of IFNγ and bone marrow transplantation of bone marrow deficient in IFNγ have also been reported. IFNγ-deficient LDLR^−/−^ mice generated by crossing LDLR^−/−^ mice with IFNγ^−/−^ mice exhibit reduced atherosclerosis (81) whilst LDLR^−/−^ chimeric mice generated by irradiating LDLR^−/−^ mice and transfer of bone marrow from crossing IFNγ^−/−^ mice exhibited aggravated atherosclerosis (82).

In summary, we have shown that congenital global B cell deficiency in μMT^−/−^ApoE^−/−^ mice decreases atherosclerosis by reducing accumulation of macrophages and CD4 T cells. When B2 cells were adoptively transferred into μMT^−/−^ApoE^−/−^ mice, we found augmented atherosclerosis. Our findings suggest that B cell recruitment of CD4 T cells contributes to atherosclerosis development. Interaction between B and CD4 T cells may be important in atherosclerosis pathogenesis and targeting B and CD4 T cell interaction may be important therapeutic target to limit arterial inflammation in atherosclerosis.

## Data Availability Statement

The datasets generated for this study are available on request to the corresponding author.

## Ethics Statement

This animal study was reviewed and approved by the Animal ethic committee of Alfred Research Alliance.

## Author Contributions

B-HT, AB, and TK conceived and designed experiments. TK, CT, PK, HH, and AC performed experiments and analyzed data. TK drafted and TK, B-HT, and AB wrote the manuscript. All authors participated and contributed in manuscript discussion and approved final manuscript.

### Conflict of Interest

The authors declare that the research was conducted in the absence of any commercial or financial relationships that could be construed as a potential conflict of interest.

## Supplementary Material

Figure S1 Genotype and phenotype of μMT^−/−^ ApoE^−/−^ mice. Genomic DNAs extracted from μMT^−/−^ ApoE^−/−^ and ApoE^−/−^ mice were subjected to polymerase chain reactions (PCRs) using appropriate primers (see section Materials and Methods for details). PCR products were separated and visualized on ethidium-stained TAE agarose gel. Representative PCR results for **(A)** μMT and **(B)** ApoE genotypes shown. Line 1- DNA ladder ΦX174 DNA/HaeIII, Line 2- μMT^−/−^ ApoE^−/−^, Line 3- μMT^−/−^ ApoE^−/−^, Line 4- μMT^+/−^ ApoE^+/−^, Line 5- μMT^+/−^ ApoE^+/−^, Line 6- μMT^+/+^ ApoE^+/+^. **(C)** Representative histograms show deficiency of CD19^+^ B cells, assessed by FACS analysis, in peripheral blood, lymph nodes, spleen, and peritoneal cavities. *n* = 12–15 per group. Representative photomicrographs and FACS histograms from three different experiments.

Figure S2 Plasma lipid profile in hyperlipidemic ApoE^−/−^ μMT^−/−^ ApoE^−/−^ mice. ApoE^−/−^ and μMT^−/−^ ApoE^−/−^ mice (male 6–8 week-old) were fed a high fat diet for 8 weeks. Plasma lipid determination was carried out at the end of experiment. Data presented as mean ± SEM of two to three independent experiments. *n* = 12–15 per group.

Figure S3 B cell deficiency results in absence of IgG and IgM in plasma and of Ig deposits in lesions. At the completion of 8 week high fat diet feeding, plasma and spleens from ApoE^−/−^ and μMT^−/−^ ApoE^−/−^ mice were collected. Plasmas were used to determine the immunoglobulins and frozen section from OCT-embedded spleens were stained with various antibodies. **(A,B)** Representative fluorescent microimages of atherosclerotic lesions stained with FITC-conjugated anti-B220 antibody and counterstained with DAPI showing that B cells are completely absent in spleens in μMT^−/−^ ApoE^−/−^ mice. ELISA determination showed **(C)** plasma total immunoglobulins (total, IgG and IgM) and **(D)** MDA-specific oxLDL-immunoglobulins (total, IgG and IgM) in ApoE^−/−^ mice but not in μMT^−/−^ ApoE^−/−^ mice. **(E)** Representative microimages of immunoglobulin deposits in atherosclerotic lesions show immunoglobulin deposits in wildtype but not in μMT^−/−^ ApoE^−/−^ mice. Data were presented as mean ± SEM of two to three independent experiments. *n* = 12–15 per group, ^*^*p* < 0.05, □ ApoE^−/−^ mice ■ μMT^−/−^ ApoE^−/−^ mice.

Figure S4 Isolation of naïve B cells for adoptive transfer. Naïve B2 cells were isolated from different donor mice using magnetic B cell isolation kit (Miltenyi Biotec). Using biotin-conjugated antibody cocktail against CD43, CD4, and Ter119, non-B2 cells such as T cells, macrophages and dendritic cells as well as activated B cells and B1a cells were positively labeled. After manual separation using MS columns, unlabelled cells were collected. Cell preparation before magnetic labeling, positively-labeled cells (positive fraction) and unlabelled cells (negative fraction) were stained with antibodies against CD19 and CD5 and FACS analysis was carried out on BD FACSCanto II (BD Biosciences). Encashment of naïve B2 cells was always >99%.

Figure S5 Plasma lipid profile of hyperlipidemic μMT^−/−^ ApoE^−/−^ mice in transfer study. B cell-deficient μMT^−/−^ ApoE^−/−^ mice (male 6–8 week-old) were adoptively transferred with naïve B2 cells, followed by 8 week HFD feeding. Plasma lipid determination was carried out at the end of experiment. Data presented as mean ± SEM of two to three independent experiments. *n* = 9 per group. □ PBS transfer, ■ WT B cell transfer,  MHCII^−/−^ B cell transfer, and  CD40^−/−^ B cell transfer.
