# Supplementary figures and images for: B Cell and CD4 T Cell Interactions Promote Development of Atherosclerosis
Source: Front Immunol. 2020 Jan 10;10:3046. doi: 10.3389/fimmu.2019.03046 (PMC6965321; doi:10.3389/fimmu.2019.03046)

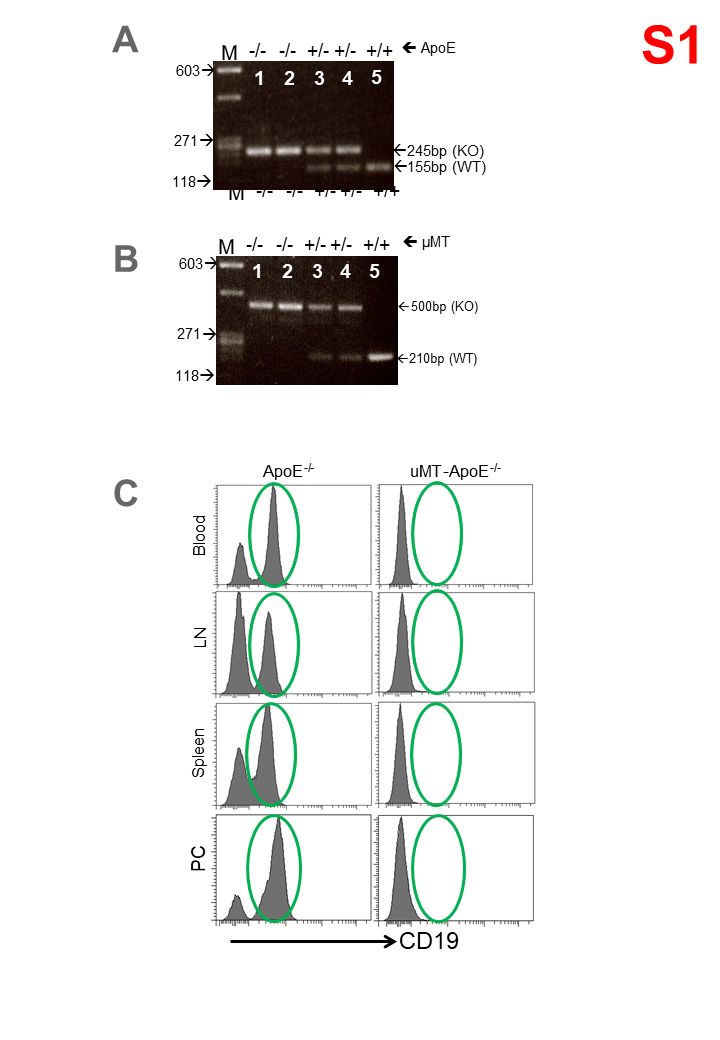

Supplement: Figure S1 — Genotype and phenotype of μMT−/− ApoE−/− mice. Genomic DNAs extracted from μMT−/− ApoE−/− and ApoE−/− mice were subjected to polymerase chain reactions (PCRs) using appropriate primers (see section Materials and Methods for details). PCR products were separated and visualized on ethidium-stained TAE agarose gel. Representative PCR results for (A) μMT and (B) ApoE genotypes shown. Line 1- DNA ladder ΦX174 DNA/HaeIII, Line 2- μMT−/− ApoE−/−, Line 3- μMT−/− ApoE−/−, Line 4- μMT+/− ApoE+/−, Line 5- μMT+/− ApoE+/−, Line 6- μMT+/+ ApoE+/+. (C) Representative histograms show deficiency of CD19+ B cells, assessed by FACS analysis, in peripheral blood, lymph nodes, spleen, and peritoneal cavities. n = 12–15 per group. Representative photomicrographs and FACS histograms from three different experiments. [file Image_1.tif]

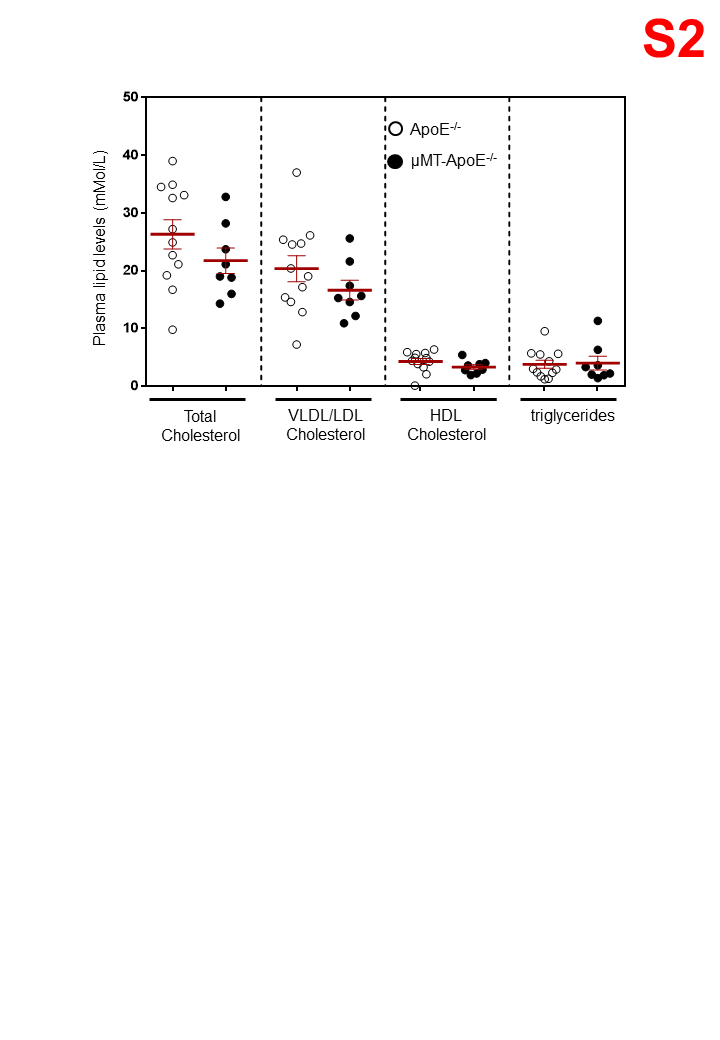

Supplement: Figure S2 — Plasma lipid profile in hyperlipidemic ApoE−/− μMT−/− ApoE−/− mice. ApoE−/− and μMT−/− ApoE−/− mice (male 6–8 week-old) were fed a high fat diet for 8 weeks. Plasma lipid determination was carried out at the end of experiment. Data presented as mean ± SEM of two to three independent experiments. n = 12–15 per group. [file Image_2.tif]

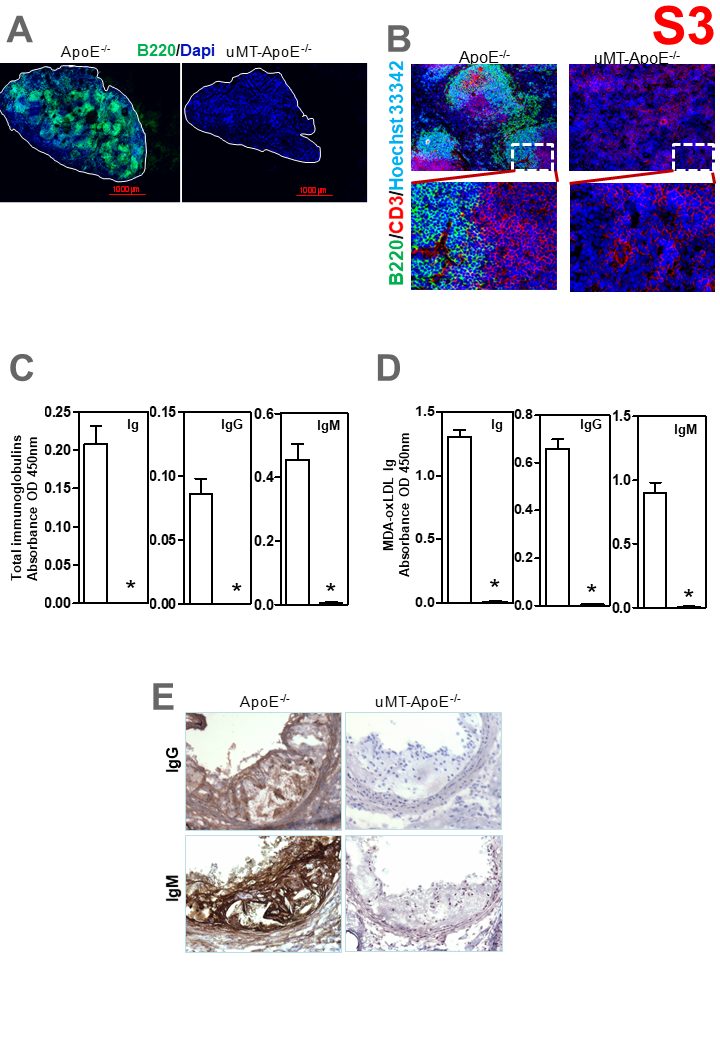

Supplement: Figure S3 — B cell deficiency results in absence of IgG and IgM in plasma and of Ig deposits in lesions. At the completion of 8 week high fat diet feeding, plasma and spleens from ApoE−/− and μMT−/− ApoE−/− mice were collected. Plasmas were used to determine the immunoglobulins and frozen section from OCT-embedded spleens were stained with various antibodies. (A,B) Representative fluorescent microimages of atherosclerotic lesions stained with FITC-conjugated anti-B220 antibody and counterstained with DAPI showing that B cells are completely absent in spleens in μMT−/− ApoE−/− mice. ELISA determination showed (C) plasma total immunoglobulins (total, IgG and IgM) and (D) MDA-specific oxLDL-immunoglobulins (total, IgG and IgM) in ApoE−/− mice but not in μMT−/− ApoE−/− mice. (E) Representative microimages of immunoglobulin deposits in atherosclerotic lesions show immunoglobulin deposits in wildtype but not in μMT−/− ApoE−/− mice. Data were presented as mean ± SEM of two to three independent experiments. n = 12–15 per group, *p < 0.05, □ ApoE−/− mice ■ μMT−/− ApoE−/− mice. [file Image_3.tif]

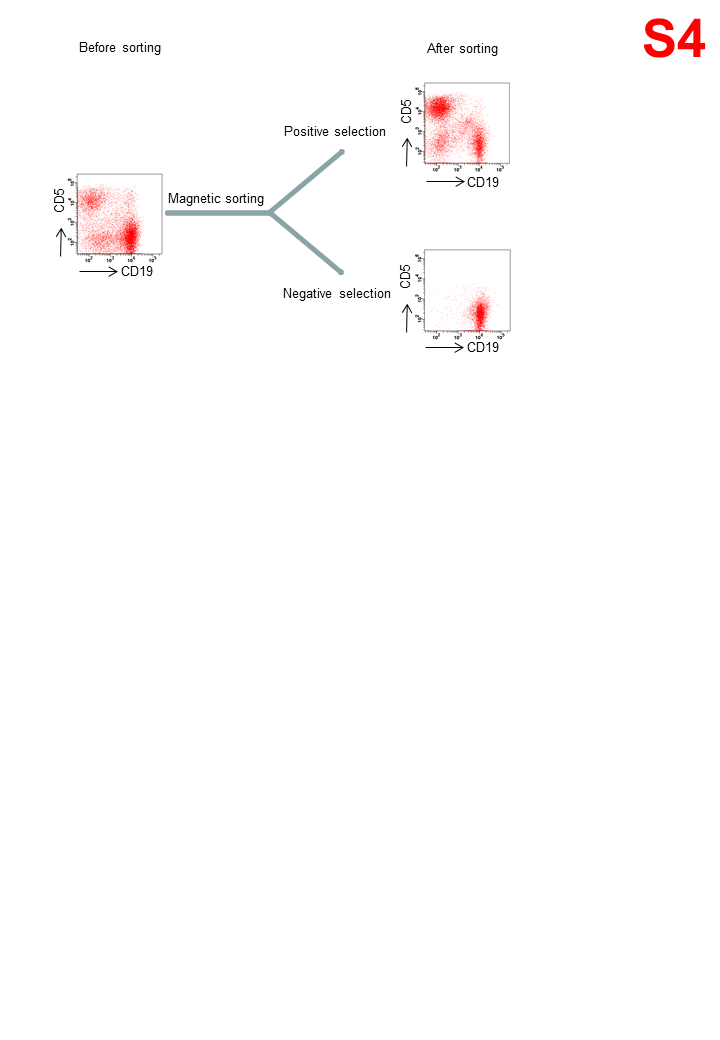

Supplement: Figure S4 — Isolation of naïve B cells for adoptive transfer. Naïve B2 cells were isolated from different donor mice using magnetic B cell isolation kit (Miltenyi Biotec). Using biotin-conjugated antibody cocktail against CD43, CD4, and Ter119, non-B2 cells such as T cells, macrophages and dendritic cells as well as activated B cells and B1a cells were positively labeled. After manual separation using MS columns, unlabelled cells were collected. Cell preparation before magnetic labeling, positively-labeled cells (positive fraction) and unlabelled cells (negative fraction) were stained with antibodies against CD19 and CD5 and FACS analysis was carried out on BD FACSCanto II (BD Biosciences). Encashment of naïve B2 cells was always >99%. [file Image_4.tif]

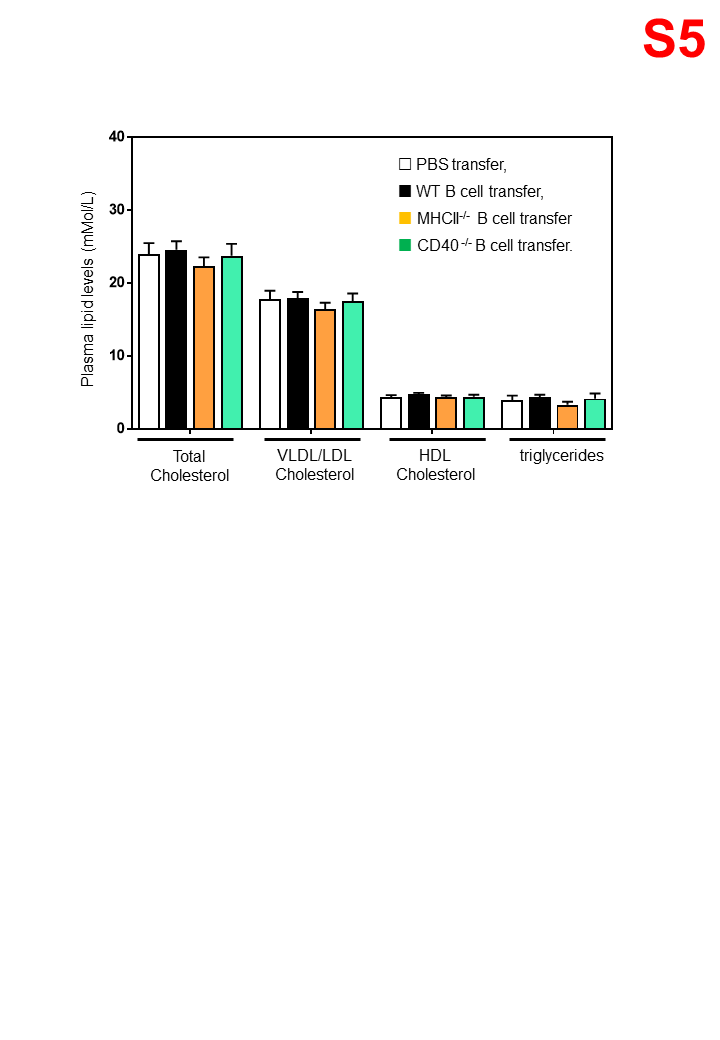

Supplement: Figure S5 — Plasma lipid profile of hyperlipidemic μMT−/− ApoE−/− mice in transfer study. B cell-deficient μMT−/− ApoE−/− mice (male 6–8 week-old) were adoptively transferred with naïve B2 cells, followed by 8 week HFD feeding. Plasma lipid determination was carried out at the end of experiment. Data presented as mean ± SEM of two to three independent experiments. n = 9 per group. □ PBS transfer, ■ WT B cell transfer, MHCII−/− B cell transfer, and CD40−/− B cell transfer. [file Image_5.tif]
